# A Left Ventricular Mechanical Dyssynchrony-Based Nomogram for Predicting Major Adverse Cardiac Events Risk in Patients With Ischemia and No Obstructive Coronary Artery Disease

**DOI:** 10.3389/fcvm.2022.827231

**Published:** 2022-03-18

**Authors:** Han Zhang, Kuangyu Shi, Mengyu Fei, Xin Fan, Lu Liu, Chong Xu, Shanshan Qin, Jiajia Zhang, Junpeng Wang, Yu Zhang, Zhongwei Lv, Wenliang Che, Fei Yu

**Affiliations:** ^1^Department of Nuclear Medicine, Shanghai Tenth People’s Hospital, Tongji University School of Medicine, Shanghai, China; ^2^Institute of Nuclear Medicine, Tongji University School of Medicine, Shanghai, China; ^3^Department of Nuclear Medicine, University of Bern, Bern, Switzerland; ^4^Department of Informatics, Technical University of Munich, Munich, Germany; ^5^Department of Radiology, Shanghai Tenth People’s Hospital, Tongji University School of Medicine, Shanghai, China; ^6^Department of Cardiology, Shanghai Tenth People’s Hospital, Tongji University School of Medicine, Shanghai, China

**Keywords:** LVMD, D-SPECT, INOCA, predict, nomogram

## Abstract

**Background:**

The risk stratification of patients with ischemia and no obstructive coronary artery disease (INOCA) remains suboptimal. This study aims to establish a left ventricular mechanical dyssynchrony (LVMD)-based nomogram to improve the present situation.

**Methods:**

Patients with suspected coronary artery disease (CAD) were retrospectively enrolled and divided into three groups: normal (stenosis <50%, without myocardial ischemia), INOCA (stenosis <50%, summed stress score >4, summed difference score ≥2), and obstructive CAD (stenosis ≥50%). LVMD was defined by ROC analysis. INOCA group were followed up for the occurrence of major adverse cardiac events (MACEs: cardiovascular death, non-fatal myocardial infarction, revascularization, stroke, heart failure, and hospitalization for unstable angina). Nomogram was established using multivariate Cox regression analysis.

**Results:**

Among 334 patients (118 [35.3%] INOCA), LVMD parameters were significantly higher in INOCA group versus normal group but they did not differ between obstructive CAD groups. In INOCA group, 27 (22.9%) MACEs occurred during a 26-month median follow-up. Proportion of LVMD was significantly higher with MACEs under both stress (63.0% vs. 22.0%, *P* < 0.001) and rest (51.9% vs. 20.9%, *P* = 0.002). Kaplan–Meier analysis revealed significantly higher rate of MACEs (stress log-rank: *P* = 0.002; rest log-rank: *P* < 0.001) in LVMD patients. Multivariate Cox regression analysis showed that stress LVMD (HR: 3.82; 95% CI: 1.30–11.20; *P* = 0.015) was an independent predictor of MACEs. The internal bootstrap resampling approach indicates that the C-index of nomogram was 0.80 (95% CI: 0.71–0.89) and the AUC values for 1 and 3 years of risk prediction were 0.68 (95% CI: 0.46–0.89) and 0.84 (95% CI: 0.72–0.95), respectively.

**Conclusion:**

LVMD-based nomogram might provide incremental prognostic value and improve the risk stratification in INOCA patients.

## Introduction

Patients presenting with the evidence of ischemia in the absence of obstructive coronary artery disease (CAD) are prevalent and increasing in frequency (1, 2). Traditionally, the presence of severe coronary stenosis has been interpreted as the most common cause of myocardial ischemia (3). Nonetheless, due to the coronary microvascular dysfunction (CMD) or vascular spasm, ischemia may also occur in non-obstructive CAD (stenosis <50%), which was recently termed as INOCA (4). Since the primary prevention of cardiovascular risk score for asymptomatic people underestimated the risk, the prognosis was once considered to be benign (5–7). Conversely, evidence indicates that patients with INOCA are at more elevated risk for future major adverse cardiac events (MACEs) (8), yet not a well-established reliable approach to predict MACEs (9).

Left ventricular mechanical dyssynchrony (LVMD) is defined as the differences in the timing of contraction or relaxation among myocardial segments (10, 11), and it can be well assessed by myocardial perfusion imaging (MPI) phase analysis. Considerable evidence confirmed that LVMD has shown significant prognostic value in many cardiovascular events caused by severe coronary stenosis, such as heart failure (12), ventricular arrhythmias (13), and dilated cardiomyopathy (14). Nonetheless, at present, the prognostic value of LVMD in patients with INOCA remains unknown.

The new scanning device D-SPECT, equipped with new solid-state cadmium zinc telluride detectors, directly converts radiation into electric signals, allowing an improvement in terms of image accuracy and acquisition time (15). Moreover, related to the higher spatial resolution, D-SPECT can better delineate myocardial walls to accurately evaluate the LVMD parameters (16).

Accordingly, the goal of this study was to determine the prognostic value of LVMD evaluated using D-SPECT in patients with INOCA and construct an LVMD-based nomogram to improve the risk stratification.

## Materials and Methods

### Study Population

We retrospectively analyzed the patients who underwent stress-rest MPI and coronary angiography during the same month in Shanghai Tenth People’s Hospital from 2017 to 2020. Among the 486 patients, 152 were excluded based on the following criteria:

1. Previous percutaneous coronary interventions or myocardial infarction (*n* = 76)
2. Cardiac resynchronization therapy (*n* = 5) and cardiac pacemaker (*n* = 3)
3. Severe intestinal interference in MPI (unable to delineate the endocardium) (*n* = 25)
4. Drug stress test terminated early (*n* = 17)
5. Heart failure [left ventricular (LV) ejection fraction (LVEF) <35%] (*n* = 10)
6. Too small left ventricle leads to high EF value (end-systolic volume (ESV) <10 ml, end-diastolic volume (EDV) <30 ml, LVEF >90%) (*n* = 16).

**FIGURE 1:**
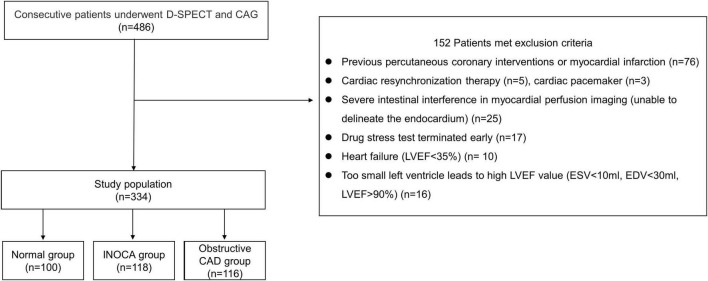
Patient flowchart.

A total of 334 patients were included in the final study cohort. We divided them into three groups: (1) normal group (each coronary artery stenosis <50%, without myocardial ischemia), (2) INOCA group (each coronary artery stenosis <50%, with myocardial ischemia, summed stress score (SSS) >4 and summed difference score (SDS) ≥2), (3) obstructive CAD group (any coronary artery stenosis ≥50%). The patient flowchart is shown in Figure 1.

The hospital ethics committee approved this retrospective study and informed consent was waived in all patients (No. SHSY-IEC-4.1/21-289/01) and registration by the Chinese Clinical Trials Registry (ChiCTR2000037112).

### Image Acquisition Protocol

Equipment: D-SPECT^®^ Cardiac System Model 003 (Spectrum Dynamics Medical Ltd., Israel, Caesarea, Serial No. 5217).

Imaging drugs: ^99*m*^Tc-MIBI (Shanghai Xinke Pharmaceutical Co., Ltd., Shanghai, China).

Stress test drugs: Adenosine injection (Penglai Nuokang Pharmaceutical Co., Ltd., Penglai, Shandong Province, China).

Scanning protocol: stop taking β-blockers, theophylline, dipyridamole, and ACEI drugs for at least 12 h before examination. The patient was first injected with ^99*m*^Tc-MIBI 10 mCi (370MBq) intravenously. After 30 min, the fat meal was eaten to eliminate intestinal disturbance. After 1 h rest, MPI was performed. Each detector column is independently rotated 110° along its long axis, collecting data from both the patient’s seat and the supine position, focusing on a pre-specified region of interest (ROI) including the heart. A pre-scan of 30–60 s is required before the acquisition to define the ROI. Around 30 min after the end of the rest scan, the patient underwent an drug stress test, and adenosine was instilled at a rate of 140 μg/(kg⋅min) through the venous access. At the same time, adenosine should be injected for ^99*m*^Tc-MIBI 25 mCi (925 MBq) at the end of 3 min of infusion, and then adenosine should be continued for 3 min. Heart rate and blood pressure were monitored and recorded before the injection of adenosine, 3 min of injection, the end of the injection, and 3 min after the end of the injection. After the end of the drug test, stress MPI was performed in the same condition.

### Image Analysis

D-SPECT images were independently interpreted by two experienced nuclear cardiologists blinded to the patient characteristics. According to the 17-segment method, the semi-quantitative 4-point method is used for evaluation: 0 points = normal; 1 point = mild reduction; 2 points = severe reduction; and 3 points = defect. Quantitative gated SPECT image processing software was used to calculate LV EDV, LV ESV, LVEF, SSS, summed rest score (SRS), SDS, phase bandwidth (PBW), phase standard deviation (PSD), and ENTROPY. Myocardial ischemia was considered positive when the SSS >4 and SDS ≥2 (17).

### Follow-Up

Patients with INOCA were followed up every 6 months through telephonic enquiries and hospital history records collected by the cardiologist. The median follow-up time was 26 months, which is defined as the D-SPECT examination date to the follow-up date.

The primary MACEs endpoint include cardiovascular death, non-fatal myocardial infarction, non-fatal stroke, heart failure, coronary revascularization, or hospitalization for unstable angina (18). The patient’s first event was considered as a MACEs event, and the time calculated for survival analysis is defined as the time from the date of inclusion to the occurrence of the first event.

### Statistical Analysis

Continuous data were presented as mean ± standard deviation, and categorical data were presented as frequency and percentage. The χ^2^ test was used for categorical data. One-way ANOVA or Kruskal–Wallis test was used for the three groups’ analysis, while Bonferroni test or Tamhane’s T2 test was used for *post hoc* analysis. Independent samples *t-*test was used to compare the means of continuous variables between the MACEs group and non-MACEs group. The receiver operating characteristic (ROC) curve was used to calculate the best cut-off value of LVMD for clinical MACEs prediction. The cumulative incidence of MACEs was estimated using the Kaplan-Meier method and compared by the log-rank test. All variables were first assessed by univariate Cox proportional hazards regression analysis. Baseline variables that were considered clinically relevant or that showed a univariate relationship with outcome were entered into multivariate Cox proportional hazards regression model (19). Results were presented as hazard ratio (HR) and 95% confidence intervals (95% *CIs*). A two-sided *P*-value of <0.05 was considered significant. All statistical analyses were performed using SPSS 16.0 for Windows (SPSS Inc., Chicago, IL, United States) and MedCalc 18.0.

### Nomogram Construction and Evaluation

The final factors for the nomogram were identified by multivariate Cox proportional hazards regression model using a threshold *P*-value of <0.05. Bootstraps of 1,000 resamples were set. Discrimination was evaluated by the Harrell concordance index (C-index) and AUC value. Calibration was measured using the calibration curve and Hosmer-Lemeshow test. Decision curve analysis (DCA) was performed to assess the clinical usefulness of the nomogram in the whole cohort. Nomogram was constructed using R (version 3.6.0; R Foundation for Statistical Computing) with RStudio (version 1.0.136).

## Results

### Patients’ Characteristics

A total of 334 patients (mean age: 62.6 ± 10.2 years; 192 [57.5%] men) were included in the final cohort. Compare to the normal group (42 [42.0%] men), INOCA group (66 [55.9%] men) have a higher proportion of men, a higher height, and a lower proportion of normal coronary arteries (0% stenosis). Moreover, INOCA group was more commonly treated with Stains. Besides, compared with INOCA group, obstructive CAD groups (84 [72.4%] men) had higher proportion of men and 2-vessel and 3-vessel diseases and most frequently received aspirin, statins, and β-blockers medications. Baseline characteristics are given in Table 1.

**TABLE 1:** Clinical characteristics of the study population (*n* = 334).

	Normal (1) (*n* = 100)	INOCA (2) (*n* = 118)	*P-*value (1 vs 2)	CAD (3) (*n* = 116)	*P-*value (2 vs 3)
**Patient characteristics**					
Age, years	63.46 ± 10.84	61.85 ± 9.36	0.24	62.52 ± 10.53	0.61
Male gender, n (%)	42(42.0%)	66(55.9%)	0.04*	84(72.4%)	<0.01*
Height, cm	163.32 ± 8.34	166.74 ± 8.03	0.002*	167.68 ± 7.64	0.36
Weight, Kg	65.99 ± 12.23	68.70 ± 10.58	0.08	69.99 ± 10.36	0.35
Body mass index, kg/m^2^	24.60 ± 3.09	24.70 ± 3.18	0.82	24.88 ± 3.09	0.66
Hypertension, n (%)	65(65.0%)	73(61.9%)	0.63	77(66.4%)	0.47
Diabetes, n (%)	18(18.0%)	22(18.6%)	0.90	33(28.4%)	0.08
Dyslipidaemia, n (%)	35(35.0%)	33(28.0%)	0.26	36(31.0%)	0.61
Current smoker, n (%)	18(18.0%)	25(21.2%)	0.56	30(25.9%)	0.40
**Baseline medications**					
Aspirin, n (%)	47(47.0%)	59(50.0%)	0.66	90(77.6%)	<0.001*
Statins, n (%)	64(64.0%)	92(78.0%)	0.02*	106(91.4)	0.004*
Beta blockers, n (%)	43(43.0%)	46(39.0%)	0.55	62(53.4%)	0.03*
CCB, n (%)	42(42.0%)	42(35.6%)	0.33	48(41.4%)	0.36
ACEI or ARB, n (%)	21(21.0%)	22(18.6%)	0.66	25(21.6%)	0.60
**Angiographic findings**					
LAD	61	83		100	
LCX	18	25		61	
RCA	26	43		76	
0-vessel, n (%)	31(31.0%)	18(15.3%)	0.006*	0(0%)	<0.001*
1-vessel, n (%)	43(43.0%)	59(50.0%)	0.30	38(32.7%)	0.007*
2-vessels, n (%)	16(16.0%)	31(26.3%)	0.07	35(30.2%)	0.51
3-vessels, n (%)	10(10.0%)	10(8.5%)	0.70	43(37.1%)	<0.001*

The bolded values and * both represent P < 0.05.

### Left Ventricular Functions and Left Ventricular Mechanical Dyssynchrony Parameters

Compared to the normal group, under both stress and rest, INOCA group had worse LV function parameters that include LVEF (*P* = 0.005, *P* = 0.030), SSS (*P* < 0.001), SRS (*P* < 0.001), SDS (*P* < 0.001), ESV (*P* < 0.001, *P* < 0.001), EDV (*P* = 0.007, *P* < 0.001), PBW (*P* = 0.005, *P* = 0.001), PSD (*P* = 0.005, *P* = 0.002), and ENTROPY (*P* = 0.001, *P* < 0.001).

**FIGURE 2:**
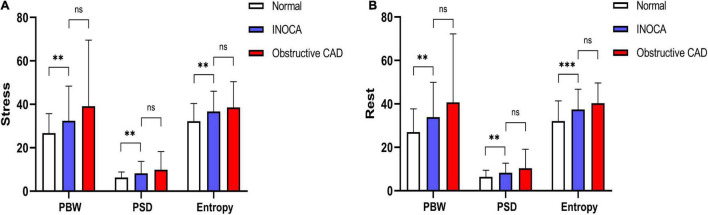
**(A)** Stress LVMD parameters between three groups. **(B)** Rest LVMD parameters between three groups (^∗^*P* < 0.05, ^∗∗^*P* < 0.01, and ^∗∗∗^*P* < 0.001).

Compared to the obstructive CAD group, total perfusion scores such as SSS (*P* < 0.001), SRS (*P* < 0.001) and SDS (*P* < 0.001) in the INOCA group were lower, and the rest of the LVEF were higher (*P* = 0.02). The differences between other indicators were not statistically significant. All results are summarized in Figure 2 and Table 2.

**TABLE 2:** LV functions of the study population.

	Normal (1) (*n* = 100)	INOCA (2) (*n* = 118)	*P-*Value (1 vs 2)	CAD (3) (*n* = 116)	*P-*value (2 vs 3)
**Stress**					
LVEF	67.94 ± 8.85	63.39 ± 11.98	0.005*	60.14 ± 13.86	0.16
PER	−3.56 ± 0.64	−3.23 ± 0.76	0.002*	−3.13 ± 0.87	0.59
PFR	2.48 ± 0.73	2.33 ± 0.77	0.40	2.16 ± 0.69	0.21
SSS	0.00 ± 0.00	5.0(4.0–7.0)	<0.001*	7.0(5.0–10.0)	<0.001*
ESV	21.13 ± 10.62	31.32 ± 25.40	<0.001*	37.59 ± 30.29	0.24
EDV	63.07 ± 19.06	75.91 ± 32.88	0.007*	83.67 ± 36.11	0.24
PBW	26.76 ± 8.92	32.19 ± 15.78	0.005*	39.10 ± 30.43	0.09
PSD	6.33 ± 2.48	8.14 ± 5.47	0.005*	9.90 ± 8.33	0.16
ENTROPY	32.22 ± 8.14	36.53 ± 9.19	0.001*	38.53 ± 11.88	0.39
**Rest**					
LVEF	69.68 ± 11.76	65.40 ± 11.96	0.03*	60.66 ± 14.38	0.02*
PER	−3.66 ± 0.94	−3.43 ± 0.87	0.17	−3.21 ± 0.86	0.16
PFR	2.68 ± 0.92	2.37 ± 0.70	0.02*	2.16 ± 0.77	0.07
SRS	0.00 ± 0.00	1(0.0–3.0)	<0.001*	2(2.0–4.0)	<0.001*
ESV	18.44 ± 10.16	28.44 ± 24.57	<0.001*	34.22 ± 28.61	0.27
EDV	59.61 ± 19.19	73.59 ± 31.65	<0.001*	78.05 ± 34.98	0.67
PBW	27.03 ± 10.68	33.88 ± 16.02	0.001*	40.71 ± 31.50	0.09
PSD	6.46 ± 2.94	8.17 ± 4.31	0.002*	10.36 ± 8.76	0.05
ENTROPY	32.13 ± 9.23	37.22 ± 9.19	<0.001*	40.28 ± 11.90	0.09
SDS	0.00 ± 0.00	4(3.0–5.0)	<0.001*	5(3.0–6.0)	<0.001*
TID	1.09 ± 0.15	1.08 ± 0.12	0.78	1.09 ± 0.12	0.81

The bolded values and * both represent P < 0.05.

### Stress-Induced Changes in Left Ventricular Functions and Left Ventricular Mechanical Dyssynchrony Parameters

We analyzed the LV function and LVMD parameters under both stress and rest in three groups, respectively. There was no statistical difference in all indicators between the three groups of patients. All results are summarized in Table 3.

**TABLE 3:** Stress-induced changes in LV functions and LVMD parameters between three groups.

		Normal	*P-*value	INOCA	*P-*value	CAD	*P-*value
LVEF	Stress	67.94 ± 8.85	0.24	63.39 ± 11.98	0.20	60.14 ± 13.86	0.78
	Rest	69.68 ± 11.76		65.40 ± 11.96		60.66 ± 14.38	
PER	Stress	−3.56 ± 0.64	0.35	−3.23 ± 0.76	0.06	−3.13 ± 0.87	0.45
	Rest	−3.66 ± 0.94		−3.43 ± 0.87		−3.21 ± 0.86	
PFR	Stress	2.48 ± 0.73	0.10	2.33 ± 0.77	0.67	2.16 ± 0.69	0.93
	Rest	2.68 ± 0.92		2.37 ± 0.70		2.16 ± 0.77	
ESV	Stress	21.13 ± 10.62	0.07	31.32 ± 25.40	0.38	37.59 ± 30.29	0.38
	Rest	18.44 ± 10.16		28.44 ± 24.57		34.22 ± 28.61	
EDV	Stress	63.07 ± 19.06	0.20	75.91 ± 32.88	0.58	83.67 ± 36.11	0.23
	Rest	59.61 ± 19.19		73.59 ± 31.65		78.05 ± 34.98	
PBW	Stress	26.76 ± 8.92	0.85	32.19 ± 15.78	0.50	39.10 ± 30.43	0.69
	Rest	27.03 ± 10.68		33.88 ± 16.02		40.71 ± 31.50	
PSD	Stress	6.33 ± 2.48	0.73	8.14 ± 5.47	0.95	9.90 ± 8.33	0.68
	Rest	6.46 ± 2.94		8.17 ± 4.31		10.36 ± 8.76	
ENTROPY	Stress	32.22 ± 8.14	0.94	36.53 ± 9.19	0.56	38.53 ± 11.88	0.27
	Rest	32.13 ± 9.23		37.22 ± 9.19		40.28 ± 11.90	

### Follow-Up Outcomes

During a median period of 26 months (interquartile range: 17.7–35.0) of follow-up among 118 patients with INOCA, MACEs occurred in 27 patients (22.9%) who had heart failure (*n* = 6, 5.1%), had stroke (*n* = 8, 6.8%), and was hospitalized for unstable angina (*n* = 13, 11.0%).

Major adverse cardiac events group had higher BMI and frequently receive ACEI/ARB medication. Under both stress and rest, compared with non-MACES group, MACEs group had lower LVEF (*P* = 0.01, *P* = 0.02), higher SSS (*P* < 0.001), SRS (*P* < 0.001), SDS (*P* < 0.001), ESV (*P* = 0.02, *P* = 0.04), EDV (*P* = 0.03, *P* = 0.04), PBW (*P* = 0.006, *P* = 0.02), PSD (*P* = 0.013, *P* = 0.006), ENTROPY (*P* = 0.005, *P* < 0.001) (Table 4).

**TABLE 4:** Comparison of risk factors between MACEs and non-MACEs groups.

	MACEs group (*n* = 27)	Non-MACEs group (*n* = 91)	*P-*value
**Patient characteristics**			
Age, years	59.04 ± 10.23	62.69 ± 8.98	0.08
Male gender, n (%)	11(40.7%)	55(60.4%)	0.07
Height, cm	166.44 ± 7.72	166.83 ± 8.16	0.87
Weight, Kg	71.83 ± 12.43	67.77 ± 9.85	0.08
Body mass index, kg/m^2^	25.81 ± 3.13	24.37 ± 3.14	0.04*
Hypertension, n (%)	20(71.4%)	53(58.2%)	0.14
Diabetes, n (%)	2(7.4%)	20(22.0%)	0.09
Dyslipidaemia, n (%)	3(11.1%)	30(33.0%)	0.03*
Current smoker, n (%)	4(14.8%)	21(23.1%)	0.36
**Baseline medications**			
Aspirin, n (%)	14(51.9%)	45(49.5%)	0.83
Statins, n (%)	19(70.4%)	73(80.2%)	0.28
Beta blockers, n (%)	13(48.1%)	33(36.3%)	0.27
CCB, n (%)	12(44.4%)	30(33.0%)	0.27
ACEI or ARB, n (%)	13(48.1%)	9(9.9%)	<0.001*
**Stress MPI**			
EF	57.29 ± 15.04	65.21 ± 10.33	0.01*
PER	−3.09 ± 0.94	−3.27 ± 0.70	0.40
PFR	2.16 ± 1.06	2.39 ± 0.64	0.29
SSS	9(7–13)	5(4–6)	<0.001*
ESV	41.18 ± 31.13	28.39 ± 22.81	0.02*
EDV	87.74 ± 40.53	72.40 ± 29.61	0.03*
PBW	43.78 ± 26.09	28.74 ± 8.65	0.006*
PSD	11.98 ± 9.67	7.00 ± 2.48	0.013*
ENTROPY	42.09 ± 11.81	34.88 ± 7.57	0.005*
LVMD, n (%)	17(63.0%)	20(22.0%)	<0.001*
**Rest MPI**			
EF	59.78 ± 14.78	67.05 ± 10.64	0.02*
PER	−3.12 ± 0.96	−3.52 ± 0.83	0.04*
PFR	2.02 ± 0.79	2.48 ± 0.64	0.002*
SRS	4(2–6)	1(0–2)	<0.001*
ESV	38.89 ± 30.05	25.34 ± 21.94	0.04*
EDV	86.81 ± 38.33	69.67 ± 28.46	0.04*
PBW	41.55 ± 19.89	31.18 ± 13.45	0.02*
PSD	10.55 ± 5.10	7.47 ± 3.80	0.006*
ENTROPY	42.72 ± 9.74	35.60 ± 8.40	<0.001*
LVMD, n (%)	14(51.9%)	19(20.9%)	0.002*
SDS	5(4–6)	4(3–5)	<0.001*
TID	1.07 ± 0.10	1.07 ± 0.13	0.95

The bolded values and * both represent P < 0.05.

**FIGURE 3:**
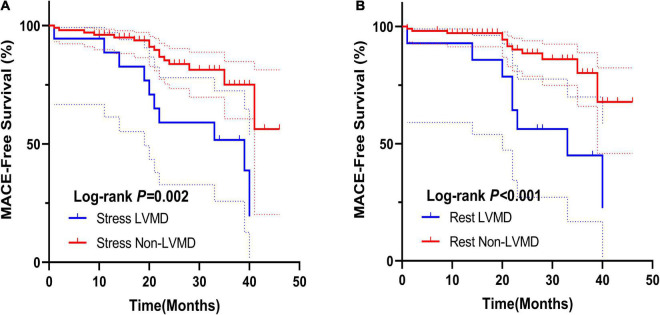
**(A)** Kaplan–Meier curves for MACEs according to stress LVMD. **(B)** Kaplan–Meier curves for MACEs according to rest LVMD.

**FIGURE 4:**
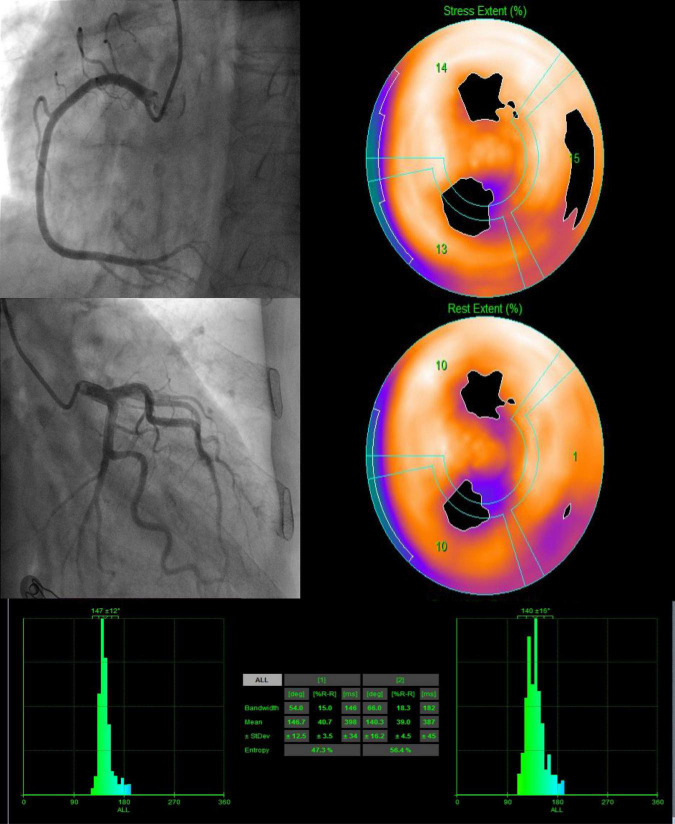
A 82-year-old female patient with INOCA (LAD stenosis 30%, SSS = 8, SDS = 4) presented LVMD under both stress and rest. After 20 months of follow-up, she was hospitalized for heart failure.

According to the cut-off value for MACEs prediction, we define stress and rest LVMD as (PBW >30°, PSD >10.1° or ENTROPY >43.3%; PBW >36°, PSD >9.4°, or ENTROPY >42.8%, respectively). Any value of PBW, PSD, or ENTROPY that exceeds the cut-off value is considered LVMD under both stress and rest. Proportion of LVMD was significantly higher with MACEs under both stress (63.0% vs. 22.0%, *P* < 0.001) and rest (51.9% vs. 20.9%, *P* = 0.002). The Kaplan-Meier MACEs-free survival analysis revealed a significantly higher rate (stress log-rank = 15.56, *P* = 0.002; rest log-rank = 7.56, *P* < 0.001) in patients with LVMD (Figure 3). In addition, one random case is used as a supplementary illustration (Figure 4).

### Predictors of Major Adverse Cardiac Events

The results of univariate and multivariable Cox regression analysis are reported in Table 5. Stress EF (HR: 0.974, 95% CI: 0.949–0.999, *P* = 0.04), SSS (HR: 1.152, 95% CI: 1.089–1.217, *P* < 0.001), stress LVMD (HR: 6.064, 95% CI: 2.684–13.69, *P* < 0.001), Rest EF (HR: 0.973, 95% CI: 0.949–0.998, *P* = 0.035), SRS (HR: 1.084, 95% CI: 1.001–1.173, *P* = 0.047), SDS (HR: 1.34, 95% CI: 1.112–1.614, *P* = 0.002), Rest PFR (HR: 0.502, 95% CI: 0.299–0.840, *P* = 0.009), Rest LVMD (HR: 2.750, 95% CI: 1.291–5.858, *P* = 0.008) were significant predictors of MACEs. In multivariable Cox regression analysis, stress LVMD (HR: 3.82, 95% CI: 1.30–11.20, *P* = 0.015), female gender (HR: 3.79, 95% CI: 1.48–9.74, *P* = 0.006), SSS (HR: 1.14, 95% CI: 1.05–1.25, *P* = 0.003), and rest PFR (HR: 0.57, 95% CI: 0.35–0.95, *P* = 0.03) resulted independent predictors of MACEs in INOCA group.

**TABLE 5:** Univariate and multivariate cox regression analysis for predicting MACEs.

	Univariate analysis		Multivariate analysis	
	HR (95%CI)	*P-*value	HR (95%CI)	*P-*value
Female	1.86(0.86–4.02)	0.12	3.79(1.48–9.74)	0.006*
Age, years	0.97(0.93–1.01)	0.17		
BMI, kg/m^2^	1.10(0.99–1.23)	0.07		
Stress EF	0.97(0.95–1.00)	0.04*		
SSS	1.15(1.09–1.22)	<0.001*	1.14(1.05–1.25)	0.003*
Stress ESV	1.01(1.00–1.02)	0.11		
Stress EDV	1.01(1.00–1.02)	0.12		
Stress LVMD	6.06(2.68–13.69)	<0.001*	3.82(1.30–11.20)	0.015*
Rest EF	0.97(0.95–1.00)	0.03*		
SRS	1.08(1.00–1.17)	0.047*		
SDS	1.34(1.11–1.61)	0.002*		
Rest PFR	0.50(0.30–0.84)	0.009*	0.57(0.35–0.95)	0.03*
Rest ESV	1.01(1.00–1.02)	0.09		
Rest EDV	1.01(1.00–1.02)	0.09		
Rest LVMD	2.75(1.29–5.86)	0.008*		

The bolded values and * both represent P < 0.05.

### Nomogram Construction and Evaluation

**FIGURE 5:**
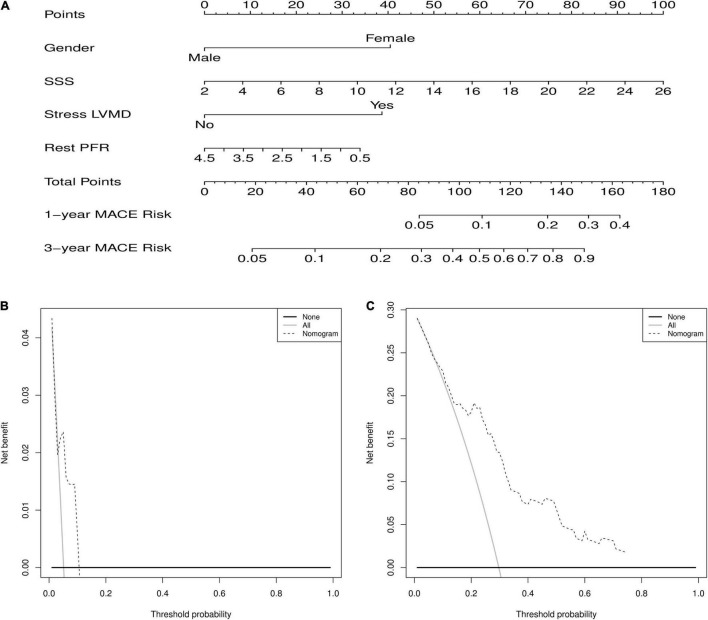
**(A)** Nomogram for predicting 1 and 3 years of MACEs risk. For individualized predictions, a vertical line is drawn upward based on the patient’s characteristics to calculate the corresponding total score. The 1-year and 3-year MACEs risk is then calculated by drawing a vertical line down from “Total Points” based on the sum. **(B)** Decision curve analysis for 1-year predict. **(C)** Decision curve analysis for 3-year predict.

**FIGURE 6:**
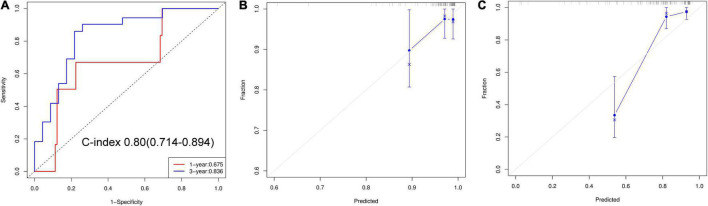
**(A)** ROC curves of nomogram for predict 1 and 3 years of MACEs risk. **(B)** Calibration curve for 1-year predict. **(C)** Calibration curve for 3-year predict.

The nomogram predicts MACEs risk based on stress LVMD, gender (female), SSS, and rest PFR (Figure 5). The C-index (0.804, 95% CI: 0.714–0.894) and AUC values for 1 and 3 years of MACEs risk were (0.675, 95% CI: 0.460–0.891) and (0.836, 95% CI: 0.720–0.951), respectively, indicating good discrimination of the nomogram. The calibration curve demonstrated good agreement between prediction and observation; moreover, the Hosmer-Lemeshow test (χ^2^ = 1.453, *P* = 0.993) suggested that there was no departure from perfect fit. The DCA demonstrated that the nomogram provided a higher net benefit across a wider reasonable range of threshold probabilities for predicting MACEs risk (Figure 6).

## Discussion

This study has three main findings. First, the LVMD parameters of INOCA patients are worse than that of the normal group, and there is no significant difference between the obstructive CAD groups. Second, stress LVMD, female gender, SSS, and rest PFR independently predicted the occurrence of MACEs. Finally, the nomogram based on the stress LVMD is used to identify high MACEs risk and improve the risk stratification among INOCA patients.

### Patient Characteristics/Left Ventricular Mechanical Dyssynchrony Parameters

Although the diagnostic criteria in INOCA patients are not yet uniform, the previous meta-analysis has shown that the incidence of all-cause death and non-fatal myocardial infarction in patients with INOCA (1.32/100 person-years) is much higher than that in patients with normal epicardial angiography (0.52/100 person-years) (8). Similarly, our results also proved that compared to the normal group many parameters reflecting the systolic and diastolic functions of INOCA in patients are impaired. We suspected that this may be caused by multifactorial mechanisms including CMD and coronary spasm, which directly cause the mismatch between blood supply and myocardial oxygen consumption in the condition of non-obstructive coronary atherosclerosis, further leading to left ventricular function damage and ventricular remodeling (20, 21).

As an early predictive marker of myocardial disease, LVMD can be measured using various imaging modalities including echocardiography, cardiac magnetic resonance imaging, and G-MPI phase analysis (22–24). Among them, G-MPI phase analysis has been widely used for the diagnosis of LVMD in patients with obstructive CAD due to its high reproducibility and good localizing ability for myocardial scars. Meanwhile, the latest advance in SPECT imaging, D-SPECT, with its ultra-fast scanning speed and good delineation of the endocardium, brings the phase analysis technology to the new stage (25). This study indicates that under both stress and rest, the phase analysis parameters in INOCA group are higher than normal group, which is related to SSS and SRS difference between the two groups. Remarkably, although CAD group showed higher SSS and SRS, the difference of phase analysis parameters wasn’t significant. A possible explanation for this might be that in addition to myocardial ischemia caused by coronary artery stenosis, chronic repetitive CMD can also lead to progressive fibrosis of the myocardium, which in turn causes a decrease in left ventricular wall motion (26). Another possible explanation is based on the previous study by Gimelli A et al.: CMD patients are usually accompanied with sympathetic activity impairment, through higher innervation heterogeneity to reduce myocardial contractility, which significantly correlated with left ventricular wall motion (27).

### Clinical Predictors

Many recent studies have demonstrated the predicted efficiency of LVMD in cardiac disease, also including the normal perfusion in non-coronary diseases. Fudim M et al. noted LVMD measured by G-MPI were associated with adverse outcomes in 1,310 patients with CAD (24). Malik D et al. reported that LVMD is a novel prognostic marker in patients with diabetes mellitus with normal perfusion and left ventricular systolic functions, which are related to the microvascular complications (28). Whereas, unlike those previous studies, we define the LVMD cut-value as ROC Youden index instead of 2SDs above the control group value mainly for better risk stratification. Our results also proved that stress LVMD was an independent predictor of MACEs in patients with INOCA and appears to provide incremental predictive value than that of rest LVMD.

Although there is no gender difference in deaths from cardiovascular disease annually (29), women are less likely to have obstructive CAD and tend to present with INOCA than men (30, 31). Despite the proportion of women in the MACEs group being higher, gender differences did not remain significant in our study, which might be due to the small sample size. Nonetheless, after adjusting for factors in multivariate analysis, female gender remains an independent risk factor for MACEs. These relationships may partly be explained by several specific pro-inflammatory markers and psychosocial stress. Schroder J et al. in their study demonstrated that inflammatory status was associated with impaired microvascular dilatation and four cardiovascular protein biomarkers were significantly associated with CMD in women (32). Meanwhile, Konst RE et al. in a cohort of 64 women with INOCA and 64 age-matched women with CAD showed that women with INOCA experience more psychosocial distress related to a higher symptom burden (33).

LV diastolic dysfunction represents an earlier step of the ischemic cascade that has been proved relevant to the severity and degree of non-obstructive and obstructive CAD (34). Therefore, there should be a strong correlation between SSS and PFR. Our result shows that both SSS and rest PFR in the INOCA group was significantly higher than in normal group, which is also an independent predictor of MACEs. Surprisingly, despite the lower SSS in INOCA group, no differences were found in PFR under both stress and rest between CAD groups. This result may be explained by the fact that similar to LVMD, cardiac sympathetic denervation will also affect LV diastolic dysfunction and the extent of regional innervation/perfusion mismatch was an independent predictor of LV diastolic dysfunction (35).

### Usefulness of Nomogram

In terms of the current deficiency in the prediction and management guidelines for INOCA patients, we constructed a nomogram to predict MACEs in patients with INOCA. Based on the above predictors, clinicians can readily and reliably evaluate 1 and 3 years of MACEs risk in patients with INOCA by simply drawing a few lines on the nomogram. To our knowledge, this is the first time that LVMD has been included in nomogram to assess the MACEs risk. By using this nomogram, it is expected that the risk stratification and early intervention of patients with INOCA can be improved.

### Limitations

This study has several limitations. First, due to the single centre, retrospective study, our nomogram was validated only internally, and further external validation is required. Second, according to the previous studies, we believe that the main pathogenesis of most patients with INOCA is CMD and further leads to LVMD, but these patients need to be determined whether CMD is present. Finally, the MACEs incidence may be underestimated because of the short follow-up period; therefore, studies with larger numbers of patients and longer follow-up times are needed to verify our findings.

## Conclusion

Stress LVMDs evaluated from D-SPECT are the novel independent predictor of MACEs in patients with INOCA. Moreover, an LVMD-based nomogram might provide incremental prognostic value and improve the risk stratification.

## Data Availability Statement

The original contributions presented in the study are included in the article/Supplementary Material, further inquiries can be directed to the corresponding authors.

## Ethics Statement

The studies involving human participants were reviewed and approved by The ethics committee of Shanghai Tenth People’s Hospital. Written informed consent for participation was not required for this study in accordance with the national legislation and the institutional requirements.

## Author Contributions

FY, WC, and ZL: conception and design. HZ and MF: data collection and analysis. KS: language editing and polishing. XF, LL, and CX: data curation. SQ, JZ, YZ, and JW: provision of study materials. All authors contributed to the article and approved the submitted version.

## Conflict of Interest

The authors declare that the research was conducted in the absence of any commercial or financial relationships that could be construed as a potential conflict of interest.

## Publisher’s Note

All claims expressed in this article are solely those of the authors and do not necessarily represent those of their affiliated organizations, or those of the publisher, the editors and the reviewers. Any product that may be evaluated in this article, or claim that may be made by its manufacturer, is not guaranteed or endorsed by the publisher.
