# Supplementary material for: A Left Ventricular Mechanical Dyssynchrony-Based Nomogram for Predicting Major Adverse Cardiac Events Risk in Patients With Ischemia and No Obstructive Coronary Artery Disease
Source: Front Cardiovasc Med. 2022 Mar 18;9:827231. doi: 10.3389/fcvm.2022.827231 (PMC8971375; doi:10.3389/fcvm.2022.827231)
Supplement: Supplementary file 1 [file Data_Sheet_1.docx]

Supplementary Material


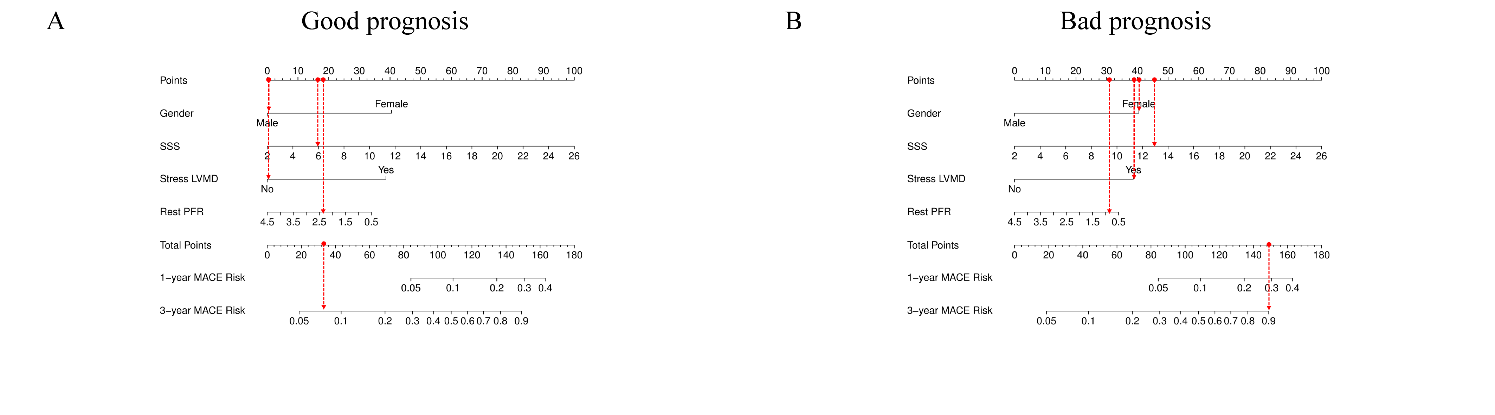


Supplementary Material 1. We have provided two examples of patients with good and bad prognosis. (A) A 59-year-old male (Gender score=0; SSS=6, score=15; stress LVMD score=0; Rest PFR=2.44, score=18; Total score 33 points), according to Nomogram 1-year MACE risk less than 5%, 3-year MACE risk between 5%-10%. (B) A 43-year-old female (Gender score=40; SSS=13, score=45; stress LVMD score=38; Rest PFR=0.78, score=30; Total score 153 points), according to Nomogram 1-year MACE risk nearly 30%, 3-year MACE risk greater than 90%.
